# Moderate Long-Term Modulation of Neuropeptide Y in Hypothalamic Arcuate Nucleus Induces Energy Balance Alterations in Adult Rats

**DOI:** 10.1371/journal.pone.0022333

**Published:** 2011-07-22

**Authors:** Lígia Sousa-Ferreira, Manuel Garrido, Isabel Nascimento-Ferreira, Clévio Nobrega, Ana Santos-Carvalho, Ana Rita Álvaro, Joana Rosmaninho-Salgado, Manuella Kaster, Sebastian Kügler, Luís Pereira de Almeida, Claudia Cavadas

**Affiliations:** 1 Center for Neuroscience and Cell Biology, University of Coimbra, Coimbra, Portugal; 2 Department of Neurology, Viral Vectors Laboratory, University Medicine Göttingen, Göttingen, Germany; 3 Department of Biology and Environment, University of Trás-os-Montes and Alto Douro, Vila Real, Portugal; 4 Faculty of Pharmacy, University of Coimbra, Coimbra, Portugal; Paris Institute of Technology for Life, Food and Environmental Sciences, France

## Abstract

Neuropeptide Y (NPY) produced by arcuate nucleus (ARC) neurons has a strong orexigenic effect on target neurons. Hypothalamic NPY levels undergo wide-ranging oscillations during the circadian cycle and in response to fasting and peripheral hormones (from 0.25 to 10-fold change). The aim of the present study was to evaluate the impact of a moderate long-term modulation of NPY within the ARC neurons on food consumption, body weight gain and hypothalamic neuropeptides. We achieved a physiological overexpression (3.6-fold increase) and down-regulation (0.5-fold decrease) of NPY in the rat ARC by injection of AAV vectors expressing NPY and synthetic microRNA that target the NPY, respectively. Our work shows that a moderate overexpression of NPY was sufficient to induce diurnal over-feeding, sustained body weight gain and severe obesity in adult rats. Additionally, the circulating levels of leptin were elevated but the immunoreactivity (ir) of ARC neuropeptides was not in accordance (POMC-ir was unchanged and AGRP-ir increased), suggesting a disruption in the ability of ARC neurons to response to peripheral metabolic alterations. Furthermore, a dysfunction in adipocytes phenotype was observed in these obese rats. In addition, moderate down-regulation of NPY did not affect basal feeding or normal body weight gain but the response to food deprivation was compromised since fasting-induced hyperphagia was inhibited and fasting-induced decrease in locomotor activity was absent.

These results highlight the importance of the physiological ARC NPY levels oscillations on feeding regulation, fasting response and body weight preservation, and are important for the design of therapeutic interventions for obesity that include the NPY.

## Introduction

Obesity and overweight are an increasing health problem associated with the risk to develop life threatening conditions such as diabetes, cardiovascular disease and cancer. The main cause of obesity in the western society is the elevated consumption of high caloric aliments and beverages, as well as decreased physical activity.

Food intake and body weight gain are centrally regulated by the hypothalamus, where arcuate nucleus (ARC) neurons have a key role sensing and integrating peripheral signals of nutrition to downstream circuits [1], [2]. ARC neurons are divided into two distinct populations acting together to regulate feeding behavior: the orexigenic NPY/AGRP (Neuropeptide Y/Agouti-Related Protein) neurons and the anorexigenic POMC/CART (Pro-OpioMelanocortin/Cocaine-and-Amphetamine-Regulated-Transcript) neurons.

Most of the hypothalamic NPY-expressing neurons are located in the ARC and project to different areas of the hypothalamus, including the paraventricular nucleus (PVN), dorsomedial hypothalamus (DMH), ventromedial hypothalamus (VMH) and lateral hypothalamic area (LH) [3]. Other sources contribute to the hypothalamic levels of NPY, such as a moderate number of NPY-expressing neurons in the DMH [4], [5]. NPY receptors are widely distributed in hypothalamic nuclei and, in particular those receiving NPY projections [6], [7]. Within the hypothalamus, NPY acts on down-stream target neurons, including neurons in the PVN and LH, to produce feeding response [6], [8], [9].

In physiological conditions, NPY neurons located in the ARC are controlled by multiple neural and peripheral signals. These signals include the hormones leptin and insulin. Administration of leptin or insulin suppresses the expression of NPY (about 0.5 to 1.5-fold decrease of ARC NPY levels) and reduces food intake in lean rats [10], [11], [12], [13] and in rodent models with elevated NPY expression, such as ob/ob mice, fasted rats or diabetic rats [14], [15], [16], [17]. In accordance, NPY neurons express the receptors for these anorexigenic signals [18], [19], [20]. Another important factor that influences NPY expression is fasting. NPY production is stimulated by negative energy conditions, such that food deprivation induces a potent up-regulation in the expression of NPY and AGRP (5–10 fold increase as compared to basal) [21], [22], [23]. Furthermore, NPY levels return to initial values within 6 to 24 hours after re-feeding [21], [24], [25]. Additionally, the production of NPY in ARC neurons is regulated by the circadian cycle. In fact, ARC NPY concentrations oscillate during the light-dark cycle [26], with a peak during the light-phase (1.25-fold increase in NPY mRNA), that precedes the onset of nocturnal feeding and a decrease to basal values during the dark-phase. Moreover, the resultant release of NPY in the PVN is higher during the same period [24], [27].

Alterations in the hypothalamic NPY levels are also associated to obesity. In average, a 3-fold increase in NPY expression in the ARC and release in the PVN is observed in rodent models of obesity, including Zucker rats [28], [29], [30] and the db/db and ob/ob mice [14], [30], [31], [32]. Interestingly, the obese Zucker rats do not show the typical peak of NPY release at the light-dark transition, neither the circadian rhythm of feeding observed in lean rats [33]. In addition, some studies also report elevated levels of hypothalamic NPY (30%, in average) in diet-induced obese mice (DIO) [34], [35]. However, other studies show a compensatory down-regulation of NPY expression in the ARC and immunoreactivity in the PVN in rats fed with high-fat diet for variable periods of time [36], [37], [38], which was related to the inhibitor effect of elevated leptin concentrations.

So far, several pharmacological and genetic studies were performed in order to better understand the role of NPY on feeding behavior and obesity. For example, central administration of NPY by i.c.v. injection or directly into the PVN induces a robust feeding response [39], [40], [41] and chronic administration of NPY produces continuous hyperphagia, leading to obesity [42], [43]. Moreover, sustained viral overexpression of NPY on hypothalamic nuclei such as the PVN, LH and DMH, results in increased food intake and body weight gain compared to controls, with rats developing obesity [44], [45], [46]. However, the extent to which these modifications are observed is different, underlying the divergent contributions of hypothalamic nuclei to energy balance regulation. In opposition, NPY deletion on knock-out mice [47] or ablation of NPY neurons in neonatal mice [48] does not significantly affect feeding or body weight. However, ablation of NPY neurons in the ARC of adult mice leads to starvation [48], [49]. Furthermore, sustained down-regulation of ARC NPY by RNA-interference inhibits re-feeding response after fasting in adult rats, but its effects on basal food consumption or body weight were not reported [46]. In conclusion, some of these studies evoke a severe activation or total ablation of the hypothalamic NPYergic system, which may not reflect the physiological oscillations of NPY owning to the nutritional condition.

In the present study, we investigated the impact of a modest but sustained modulation of NPY within the ARC neurons on food consumption and body weight gain. The ARC is a hypothalamic nucleus of particular interest, not only because it is the primary source of hypothalamic NPY, but also because ARC neurons are highly regulated by compensatory mechanism and, so far, it is not known whether viral NPY modulation in the ARC would result or not in energy balance alterations. To achieve our aim, we overexpressed and down-regulated NPY expression in the adult rat ARC, by injection of AAV vectors expressing NPY and synthetic microRNA that target NPY, respectively. Our work shows that a moderate overexpression of NPY was sufficient to induce over-feeding, sustained body weight gain and severe obesity in adult rats. While the moderate down-regulation of NPY did not affect basal feeding or normal body weight gain but impaired the response to fasting.

## Results

### *In vitro* validation of AAV vectors for overexpression and down-regulation of NPY

**Figure 1:**
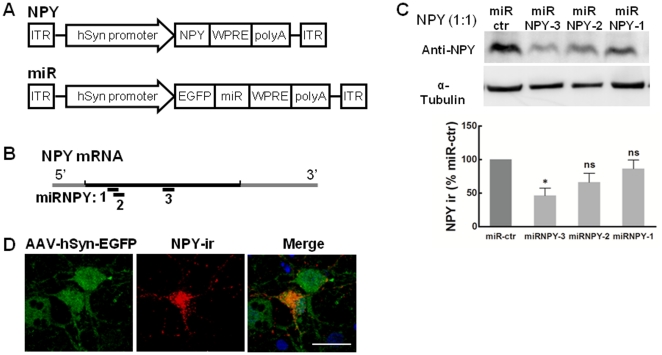
*In vitro* validation of NPY vector and synthetic microRNA for down regulation of NPY expression. (**A**) Schematic representation of the AAV vectors. The NPY expression vector contains one cassette to express NPY under a neuronal specific promoter. The microRNA expression vector contains one cassette with co-cistronic expression of EGFP and miR under the neuronal specific promoter. ITR, inverted terminal repeats; hSyn, human Synapsin promoter; WPRE, woodchuck hepatitis virus posttranslational control element; PolyA, polyadenosine sequence. Small human synapsin 1 gene promoter has shown to drive long-term neuron specific transgene expression from AAV-2 vectors [88]. (**B**) Three synthetic microRNA directed to different regions of NPY mRNA and one control microRNA (random sequence) were cloned. (**C**) Screen of microRNA targeting NPY. Representative image of NPY expression and densitometry band evaluation normalized to α-tubulin and shown as percentage of miR-ctr. Cells co-transfected with the NPY vector and one of the miR-NPY vectors, in a 1∶1 ratio. NPY expression is significantly reduced with the miR-NPY-3 vector. n = 3 independent cell transfection; * p<0.05 compared to miR-ctr control. (**D**) Transduction of NPY positive neurons (red) by AAV-hSyn-miR cassette (EGFP-green) in primary neuronal culture. Scale bar, 20 µm.

To over-express NPY in the ARC neurons we cloned the *Npy* cDNA into the AAV back-bone (Fig. 1A). To down-regulate the NPY expression we cloned three synthetic microRNAs (miR) targeting different sites of the NPY mRNA (Fig. 1B) into the AAV backbone (Fig. 1A). Additionally, we cloned one microRNA with a random sequence into the AAV backbone to be used as control (miR-ctr).

We screened the microRNAs for their efficacy to down-regulate the NPY expression by co-transfecting HEK293 cells with the pAAV-hSyn-NPY plasmid and each one of the different pAAV-hSyn-miR-NPY plasmids. NPY peptide levels were quantified by immunoblotting and densitometry band evaluation (Fig. 1C), using miR-ctr as control. The miR-NPY3 achieved the strongest NPY down-regulation (53.4±14.5% compared to miR-ctr control) and was chosen for further *in vivo* studies.

Furthermore, to validate the ability of AAV vectors to transduce and express the transgene in NPY positive neurons, we infect a rat neuronal culture with AAV vectors expressing EGFP. As seen by the co-localization of EGFP and NPY immunoreactivity (NPY-ir) in (Fig. 1D), these viral vectors can transduce the target cells (NPY-expressing neurons) and efficiently express the transgene.

### AAV-mediated moderate overexpression and down-regulation of NPY in ARC neurons of adult rats

**Figure 2:**
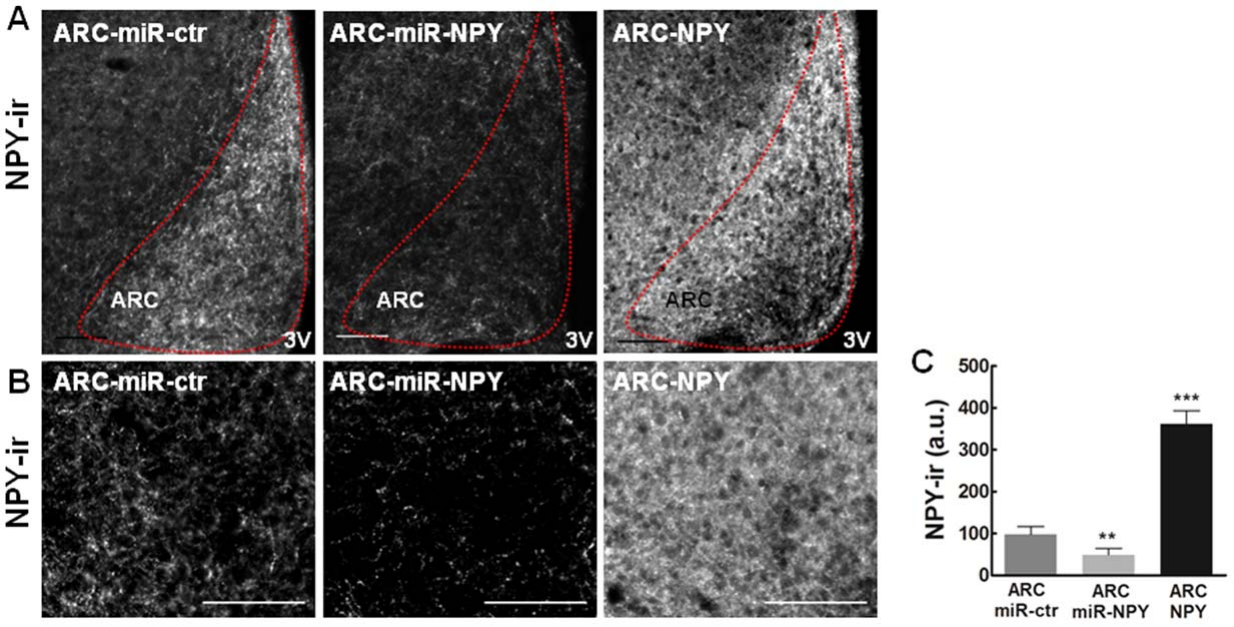
Modulation of NPY expression in the rat ARC *via* bilateral injection of AAV. (**A**) Representative images of NPY immunoreactivity (white) in the rat ARC modulated by AAV injection. Scale bar, 100 µm. (**B**) High magnification of representative images of ARC NPY immunoreactivity modulated by AAV injection. Scale bar, 100 µm. (**C**) Bilateral quantification of NPY immunoreactivity quantified through the rostro-caudal length of the rat ARC, eight weeks after AAV injection. ARC NPY immunoreactivity is 50% lower on the ARC-miR-NPY group and 3.6-fold higher on the ARC-NPY group, compared to ARC-miR-ctr group. n = 6 rats per group; ** p<0.01; *** p<0.001 compared to ARC-miR-ctr. ir, immunoreactivity.

Modulation of ARC NPY expression was achieved as shown in the representative images of NPY immunoreactivity (Fig. 2A and Fig. 2B). The levels of NPY-ir were quantified bilaterally through the rostro-caudal length of the ARC (Fig. 2C), eight weeks after AAV injection, in the following three groups: A) Control group (ARC-miR-ctr), B) ARC NPY down-regulation group (ARC-miR-NPY), and C) ARC NPY overexpression group (ARC-NPY). The NPY-ir levels were 50% lower in the ARC-miR-NPY group and 3.6-fold higher in the ARC-NPY group compared to ARC-miR-ctr group (49.0±15.7 a.u., 359.1±32.1 a.u. and 97.2±19.4 a.u., respectively). This moderate modulation of ARC NPY levels allows the development of a physiologically relevant study.

Additionally, the levels of NPY-ir were evaluated in the area surrounding the ARC to confirm that the NPY modulation was mainly restricted to the ARC. NPY-ir in the medial hypothalamic nuclei VMH/DMH were not statistically different between the three groups (p>0.05; n = 6; One-Way ANOVA). The values obtained were: ARC-miR-ctr, 124.3±4.8 a.u.; ARC-miR-NPY, 99.4±10.0 a.u.; and, ARC-NPY, 181.9±23.8 a.u.

### ARC NPY overexpression induces sustained body weight gain and severe body fat accumulation

**Figure 3:**
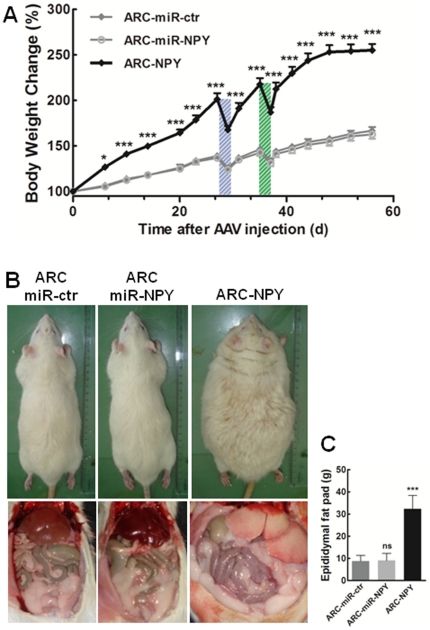
Overexpression of NPY in the ARC induces sustained body weight gain and severe fat accumulation while down-regulation of ARC NPY does not affect the body weight gain. (**A**) Cumulative body weight gains presented as percentage of initial weight in the control, ARC-miR-NPY and ARC-NPY groups. Boxes indicates food deprivation period (FD). n = 6 rats per group; * p<0.05; *** p<0.001, compared to ARC-miR-ctr group. (**B**) Representative images of rats and (**C**) epididymal fat pad weight, eight weeks after AAV injection, showing the obese phenotype and severe body fat accumulation in rats with ARC-NPY overexpression. n = 6 rats per group; ns p>0.05; *** p<0.001, compared to ARC-miR-ctr group.

We evaluated the impact of ARC NPY modulation on body weight gain (Fig. 3A). The ARC-NPY rats showed a fast and sustained increase on body weight gain, weighting 220 g more than the ARC-miR-ctr rats (351.4±43.3 g and 129.9±20.3 g of cumulative weight gain, respectively), six weeks after AAV injection; and having 88% higher body weight gain than ARC-miR-ctr group at the end of the study. In contrast, ARC-miR-NPY rats did not present significant body weight gain differences compared to control group (Fig. 3A).

Eight weeks after AAV injection, ARC-NPY rats exhibited an obese phenotype and staring coat (Fig. 3B). These rats had enlarged pale liver and excessive visceral adipose tissue deposit. Severe body fat accumulation on ARC-NPY rats was confirmed by the 266% higher epididymal fat pad weight compared to ARC-miR-ctr and ARC-miR-NPY group (32.3±6.0 g, 8.8±2.6 g and 9.1±3.3 g, respectively) (Fig. 3C).

### ARC NPY overexpression causes serum alterations consistent with obesity and elevated serum NPY

Serum analyzes were performed to evaluate possible alterations consistent with the observed obese phenotype in ARC-NPY rats. Results are summarized on Table 1. The levels of glucose, cholesterol, aspartate-amino-transferase, alanine-amino-transferase and C-reactive-protein were robustly increased in the ARC-NPY group but the triglycerides (TG) levels were not statistically different from the control group. Furthermore, the ARC-NPY group showed a severe increase on insulin and leptin compared to ARC-miR-ctr group. Interestingly, higher values of serum NPY were observed on the obese ARC-NPY group compared to the lean ARC-miR-ctr group. No significant differences were observed on serological parameters between the ARC-miR-NPY and ARC-mir-ctr groups.

**Table 1:** ARC NPY overexpression causes serum alterations consistent with obesity and elevated serum NPY.

	ARC-miR-ctr	ARC-miR-NPY	ARC-NPY
Glucose (mg/dl)	95.0±10.7	92.7±6.7^a^	397.5±118.5^c^
Cholesterol (mg/dl)	37.3±2.3	45.8±7.6^a^	233.0±16.0^c^
Triglycerides (mg/dl)	98.5±15.5	82.8±7.3^a^	121.5±21.5^a^
Aspartate-amino-transferase (IU/L)	55.0±7.0	58.8±5.3^a^	221.5±49.5^c^
Alanine-amino-transferase (IU/L)	30.5±5.5	34.5±5.5^a^	187.0±37.5^c^
C-reactive-protein (mg/dl)	7.7±0.2	7.1±0.4^a^	10.7±0.8^c^
Insulin (ng/mL)	5.5±1.9	8.1±6.6^a^	164.9±50.9^c^
Leptin (ng/mL)	9.9±3.3	11.6±7.7^a^	266.2±53.9^c^
NPY (ng/mL)	1.6±0.3	0.9±0.3^a^	3.6±0.6^b^

Serum analysis of control, ARC-miR-NPY and ARC-NPY rats, eight weeks after AAV injection. Biochemical analysis of serum from rats fed on standard chow diet. n = 5/6 rats per group for glucose, leptin and insulin and n = 4 rats per group for the other parameters.

a , p>0.5;

b , p<0.01;

c , p<0.001, compared to ARC-miR-ctr control.

### ARC NPY overexpression increases adipocytes size and decreases PPAR-gamma-2 adipogenic marker in white adipose tissue

**Figure 4:**
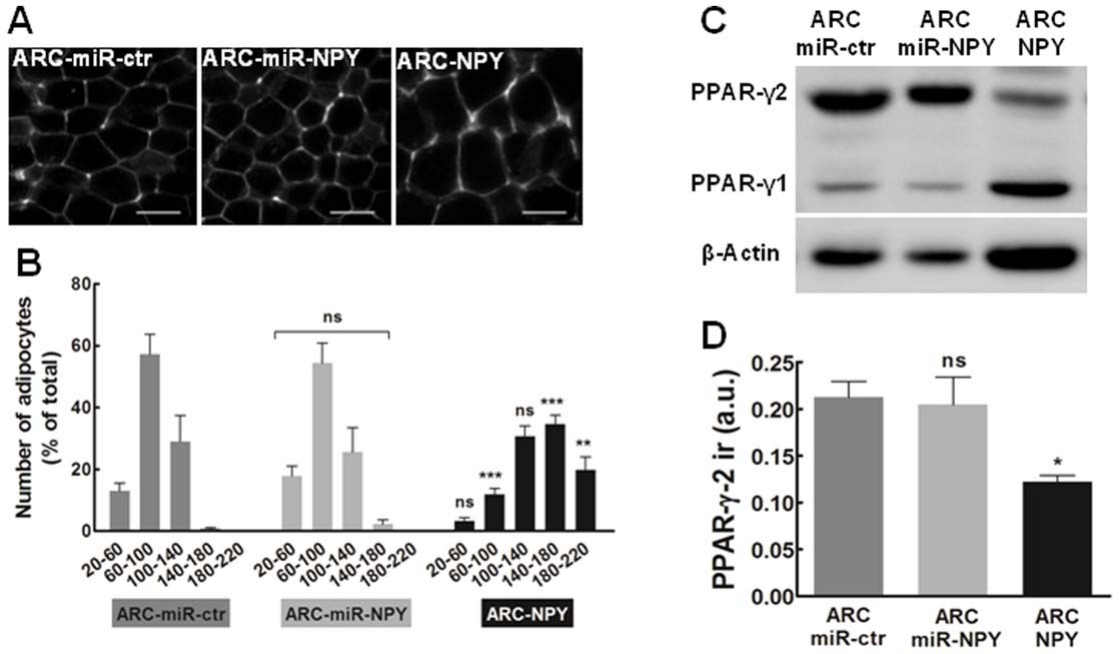
ARC NPY overexpression increases adipocytes size and decreases PPAR-gamma-2 adipogenic marker in white adipose tissue. Evaluation of adipocytes size and differentiation markers (Peroxisome Proliferator Activated Receptor type 2, PPAR-gamma-2), eight weeks after AAV injection. (**A**) Representative images of adipocytes auto-fluorescence from ARC-miR-ctr, ARC-miR-NPY and ARC-NPY groups. Scale bar, 100 µm. (**B**) Adipocytes size distribution represented as percentage of total number of adipocytes. n = 6 rats per group; ns, non-significant, ** p<0.01; *** p<0.001 compared to ARC-miR-ctr group size range. (**C**) Evaluation of PPAR-gamma isoforms in epididymal adipose tissue extracts by immunobloting. Representative images of protein level. The expression of PPAR-gamma-2 isoform is lower on ARC-NPY obese rats, compared to ARC-miR-ctr rats. (**D**) Densitometry band evaluation normalized to β-actin and shown as percentage of ARC-miR-ctr. n = 3/4 rats per group; ns p>0.5; * p<0.5, compared to ARC-miR-ctr group. ir, immunoreactivity.

We assessed possible phenotype alterations on the white adipose tissue (WAT) upon modulation of ARC NPY by evaluation of adipocytes size and differentiation marker Peroxisome Proliferator Activated Receptor type 2 (PPAR-gamma-2), eight weeks after AAV injection. Sections from epididymal fat pad were analyzed by fluorescence microscopy (Fig. 4A) and adipocytes average diameter was measured and grouped by size range (Fig. 4B). The distribution of adipocytes size was not different between the ARC-miR-ctr and ARC-miR-NPY group. However, the obese ARC-NPY group showed a higher percentage of large adipocytes and a reduced percentage of small adipocytes, compared to lean ARC-miR-ctr group (large adipocytes: range of 140–180 µm 34.6±6.3% and 0.8±0.6%, respectively and range of 180–220 µm 19.7±7.5% and 0.0%, respectively; small adipocytes: range of 60–100 µm 11.9±3.8% and 57.2±14.6%, respectively).

Furthermore, we measured PPAR-gamma protein levels in WAT extracts by immunoblotting (Fig. 4C and 4D). Unexpectedly, we observed 41% lower levels of adipogenic marker PPAR-gamma-2 on WAT from obese ARC-NPY rats compared to lean ARC-miR-ctr rats (0.12±0.01 a.u. and 0.21±0.03 a.u., respectively). No differences were observed on the PPAR-gamma-2 expression in ARC-miR-NPY group compared to control. The specificity of alteration on adipogenic PPAR-gamma-2 was confirmed by evaluation of ubiquitous PPAR-gamma-1. As expected, the levels of PPAR-gamma-1 protein were similar in the WAT samples from the three groups. These results suggest that in this obesity model there is a dysfunction in adipocytes phenotype, characterized by adipocytes with higher diameter but decreased PPAR-gamma-2 expression.

### Obesity induced by ARC NPY overexpression results from increased diurnal food intake

**Figure 5:**
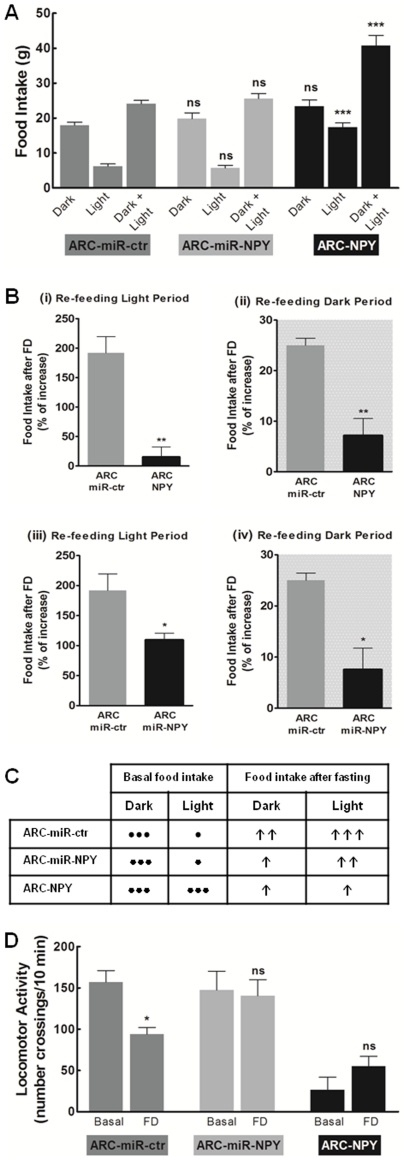
Viral modulation of ARC NPY alters feeding behavior and disrupts fasting-induced responses. (**A**) ARC NPY overexpression increases diurnal but not nocturnal food intake compared to control; whereas ARC NPY down-regulation does not change food intake compared to control. Food intake was measured on 12-hours-dark, 12-hours-light and 24-hours, six weeks after AAV injection. n = 6 rats per group. ns p>0.5; *** p<0.01 compared to ARC-miR-ctr group. (**B**) Fasting induced hyperphagia is disrupted by viral ARC NPY overexpression (**i and ii**) and viral ARC NPY down-regulation (**iii and iv**). Food intake was evaluated after 36-hours of food deprivation (FD) with re-feeding on the light (**i and iii**), and on the dark period (**ii and iv**). Data is presented as percentage of food intake increase induced by fasting, on a 12-hour period. n = 3 rats per group. * p<0.5; ** p<0.01 compared to ARC-mir-ctr group. (**C**) Table showing the effect of ARC NPY levels on basal food intake and on the increase of food intake after FD. (**D**) ARC NPY modulation impairs the locomotor activity on FD condition, five weeks after AAV injection. Data is presented as total number of crossings per 10 minutes. n = 3 rats per group. ns p>0.5; * p<0.5 compared to basal.

To test if viral modulation of NPY in the ARC would influence the normal food intake during the circadian cycle, we monitored the feeding patterns of rats during the 8 weeks following AAV injection. Six weeks after AAV injection, the ARC-miR-ctr and the ARC-miR-NPY group presented a similar food intake while the ARC-NPY group consumed 1.7-times more food a day than the control group (Fig. 5A). The overfeeding pattern presented by the ARC-NPY group was predominantly due to a 2.8-fold increase on food intake during the light period compared to ARC-miR-ctr group (17.4±2.5 g/12 hours and 6.2±1.5 g/12 hours, respectively); while the food intake observed on the dark period was not significantly different from the ARC-miR-ctr control. Therefore, modest sustained overexpression of NPY in the ARC is sufficient to induce over-feeding and abolish the normal daily feeding pattern.

### AAV-mediated modulation of ARC NPY disrupts fasting-induced responses

To further investigate the regulation of feeding upon viral ARC NPY modulation, we tested the fasting-induced hyperphagia with re-feeding in the beginning of the nocturnal and diurnal periods (Fig. 5B). The control ARC-miR-ctr group presented the typical fasting induced hyperphagia with 200% increase on food intake upon re-feeding on the light period, compared to basal (16.2±0.6 g/12 hours and 5.6±0.5 g/12 hours, respectively) and 25.0% increase on food intake upon re-feeding on the dark period, compared to basal (26.1±2.0 g/12 hours and 20.9±1.9 g/12 hours, respectively). Conversely, ARC-NPY group did not had a significant increase on food intake upon re-feeding on the light period compared to control group (15±21.3% of basal and 192±35.3% of basal, respectively) (Fig. 5B-i); however these rats already had elevated food consumption on the diurnal period (Fig. 5A). Moreover, the fasting-induced hyperphagia showed by ARC-NPY rats upon re-feeding on the dark period was significantly lower when compared to control group hyperphagia (7.1±4.2% of basal and 25.0±1.8% of basal, respectively) (Fig. 5B-ii), despite the normal basal food consumption on the dark period (Fig. 5A). These evidences suggest that sustained overexpression of NPY in ARC neurons interrupts the feeding sensing mechanism.

Fasting-induced hyperphagia was inhibited by the down-regulation of ARC NPY in the ARC-miR-NPY rats. In fact, these animals exhibited only 56% of the increase on food intake observed in control animals upon re-feeding the light period (9.5±0.6 g/12 hours after FD and 16.2±0.6 g/12 hours after FD, respectively) (Fig. 5B-iii). And 30% of the response observed in the control group upon re-feeding on the dark period (7.6±5.5% of basal and 25.0±1.8% of basal, respectively) (Fig. 5B-iv), indicating that fasting-induced hyperphagia is mediated by the increase in NPY levels at the ARC. The observed effects of ARC NPY modulation on fasting-induced feeding are summarized in Figure 5C.

Additionally, we evaluated the locomotor activity on satiated and food-deprived rats (Fig. 5D), on the fifth week after AAV injection. The control ARC-miR-ctr group exhibited a 40% decrease on its locomotor activity after 24 hours of FD, compared to basal (94.0±9.3 crossings/10 minutes and 157.0±18.7 crossings/10 minutes, respectively). Interestingly, on the ARC-miR-NPY group the locomotor activity level was not decreased under FD condition, compared to basal (140.3±29.1 crossings/10 minutes and 147.3±29.1 crossings/10 minutes, respectively). In contrast, the ARC-NPY group showed low locomotor activity both on basal and FD condition (26.0±19.3 crossings/10 minutes and 55.0±14.7 crossings/10 minutes, respectively). These results suggest that ARC NPY is necessary to the fasting-induced reduction of activity.

### Overexpression of ARC NPY deregulates feed-back mechanisms in the ARC

**Figure 6:**
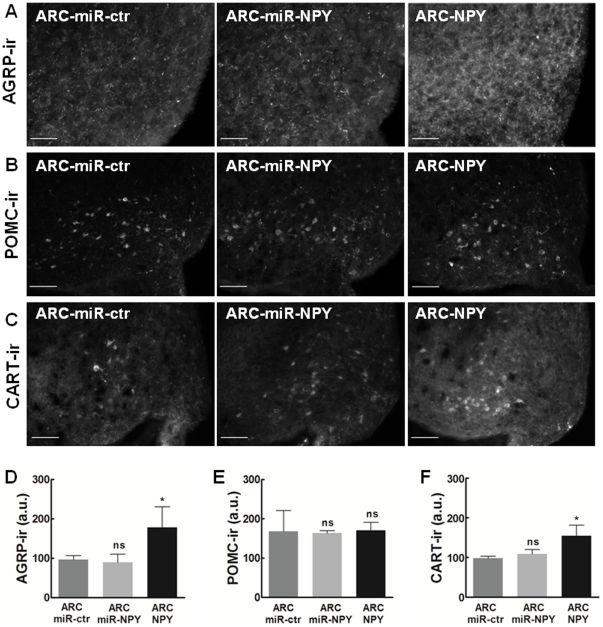
Overexpression of ARC NPY deregulates feedback mechanism in the ARC. Representative immunostaining images of ARC peptides immunoreactivity: (**A**) AGRP, (**B**) POMC and (**C**) CART. Bilateral quantification of ARC peptides immunoreactivity evaluated through the rostro-caudal length of the rat ARC, eight weeks after AAV injection: (**D**) AGRP, (**E**) POMC and (**F**) CART. Scale bar, 100 µm. n = 3/4 rats per group. ns p>0.5; * p<0.5; *** p<0.01 compared to ARC-miR-ctr. ir, immunoreactivity.

To evaluate the existence of possible compensatory mechanism caused by ARC NPY overexpression or down-regulation, we evaluated the levels of appetite-related neuropeptides in the ARC. For this purpose we quantified the immunoreactivity (ir) of AGRP, POMC and CART bilaterally in the ARC, eight weeks after AAV injection. Unexpectedly, immunohistochemical analyses for orexigenic AGRP (Fig. 6A and 6D), revealed significantly higher AGRP-ir on the ARC-NPY group, compared to ARC-miR-ctr group (166.2±35.9 a.u. and 96.6±6.9 a.u., respectively) while no differences were observed concerning AGRP-ir on the ARC-miR-NPY group (90.0±15.1 a.u.). Additionally, immunohistochemical evaluation of anorexigenic POMC (Fig. 6B and 6E), showed no differences on POMC-ir on the ARC-miR-NPY and ARC-NPY groups, compared to ARC-miR-ctr group (163.8±5.0 a.u., 170.7±16.0 a.u. and 168.2±40.9 a.u., respectively). Finally, immunohistochemical analyses for anorexigenic CART (Fig. 6C and 6D), revealed higher CART-ir on the ARC-NPY group, compared to ARC-miR-ctr control group (155.1±20.1 a.u. and 98.3±3.2 a.u., respectively) and no difference concerning CART-ir on the ARC-miR-NPY group (109.0±8.2 a.u.), compared to control. These results show that the moderate down-regulation of ARC NPY does not produce compensatory changes on ARC neuropeptides expression. Moreover, the AGRP up-regulation and absence of POMC increase are not in accordance with the elevated levels of circulating leptin suggesting a deregulation of the appetite related feed-back mechanisms in rats with ARC NPY overexpression.

## Discussion

In the present study we demonstrated that moderate long-term modulation of NPY levels in the ARC of adult rats is sufficient to affect feeding behavior and body weight gain. We showed that up-regulation of ARC NPY results in diurnal overfeeding, with disruption of feeding patterns and inhibition of fasting-induced hyperphagia. Moreover, it results in a severe obese phenotype that is not prevented by the elevated levels of circulating leptin. Additionally, we reported that modest sustained down-regulation of ARC NPY does not alter food intake on basal conditions, but inhibits fasting-induced hyperphagia. These results highlight the importance of the physiological ARC NPY levels oscillations, occurring during the circadian cycle and in response to energetic alterations, on feeding regulation and fasting-induced response, as well as in body weight preservation and, possibly, in the prevention of obesity. Overall, these findings may be important for the design of therapeutic interventions for obesity that include the NPY.

### Moderate overexpression of ARC NPY

The expression of feeding-related neuropeptides in ARC neurons is regulated by the energetic condition of the organism. In particular, the expression of orexigenic peptides NPY and AGRP is potently up-regulated by fasting (5–10 fold increase as compared to basal) [21], [22], [23]. In opposition, the levels of anorexigenic POMC are only modestly decreased with fasting (20–50% decrease as compared to basal) [50], [51]. In our study, the moderately increase on NPY levels in the ARC (3.6-fold as compared to controls) was sufficient to induce obesity, suggesting that a modest long-term increase of orexigenic peptides in the ARC is sufficient to severely alter the energy balance. Interestingly, the increase of ARC NPY levels in our model is in accordance to the up-regulation observed in genetic obesity models, such as the Zucker rats (3-fold increase in hypothalamic NPY content) [28], [29], [30]. However, it is not possible to compare it with the studies showing viral overexpression of NPY in the PVN and LH since the authors did not provide a quantification for the NPY overexpression levels [44], [52].

Nevertheless, our study shows that overexpression of NPY in ARC neurons resulted in a more severe obese phenotype than the overexpression on target neurons observed by others [44], [52]. For example, ARC-NPY rats weighted 200 g more than controls, while PVN-NPY rats weighted approximately 75 g more and LH-NPY rats weighted approximately 100 g more than controls, 50 to 60 days after the AAV injection [44], [52]. Obesity induced by NPY overexpression resulted from diurnal hyperphagia in the three models. In fact, normal rats eat approx. 6 g of food in the diurnal period but rats with ARC-NPY overexpression eat approx. 17 g of food, while rats with PVN-NPY overexpression and LH-NPY overexpression eat approx. 9 and 12.5 g of food in the same period, 40 to 50 days after the AAV injection [44], [52]. Thus, our study demonstrates that overexpression of NPY in the ARC results in a stronger hyperphagic phenotype when compared to overexpression of NPY on target neurons. Despite the fact that these divergences may be owing to technical differences including the viral titer injected and the promoter used, the severe obese phenotype observed upon ARC NPY overexpression emphasizes the importance of the ARC NPY to the control of feeding.

Sustained up-regulation of hypothalamic NPY is associated to a disruption of feeding patterns in rodent models of obesity such as Zucker rats [33], rats overexpressing NPY in the LH [44] and, as reported in this study, rats overexpressing NPY in the ARC. These observations highlight the crucial role of hypothalamic NPY oscillations during the circadian cycle to keep feeding patterns, preserve body weight and, possibly, prevent obesity in rodents. Moreover, these findings are important because deregulation of the circadian cycle in humans, as for example in nocturnal feeding, sleep loss and shift work, results in significant changes on feeding behavior and has been described as a risk factor for obesity [53], [54], [55].

In physiological conditions, the expression of ARC neuropeptides is regulated by compensatory peripheral mechanisms, including the hormone leptin, produced by adipocytes in proportional quantities to body fat storages [56], [57]. Leptin inhibits the expression of orexigenic neuropeptides NPY and AGRP [14], [15], [23] and increases the expression of POMC and CART [51], [58], [59]. Moreover, ARC NPY expression is regulated in an autocrine manner via presynaptic NPY Y_2_ receptors, present in NPY neurons and acting to decrease NPY expression [60], [61], [62].

The present study shows that a modest overexpression of NPY in the rat ARC neurons results in excessive body weight gain and fat accumulation and hyperleptinemia, demonstrating that the compensatory mechanisms regulating NPY expression are not ineffective in this condition. Moreover, several evidences in our work point to a disruption in the ability of ARC neurons to response to metabolic alterations. For example, the inhibitory effect of elevated concentrations of leptin and auto-inhibitory effect of NPY were not sufficient to restore the NPY levels. In addition, rats over-expressing NPY in the ARC did not response to fasting on the dark period, when their basal food intake was normal. And finally, the levels of ARC neuropeptides were not in accordance with the elevated levels of leptin (POMC immunoreactivity was unchanged and AGRP immunoreactivity was increased).

The fact that rats overexpressing NPY have alterations in the neuropeptides levels which are not consistent with hyperleptinemia can result from the paracrine effect of NPY on ARC neurons. Leptin acts to increase the expression of POMC [51] but, in this rat model, the elevated NPY concentrations reaching POMC neurons may be counteracting this effect. In fact, NPY and POMC neurons establish a functional network, formed mainly by dense NPY/AGRP fibers projecting to POMC cell bodies [63]. The release of NPY inhibits POMC neurons through activation of NPY Y_1_ and Y_2_ receptors expressed by POMC neurons [60], [61], [63], [64]. Moreover, NPY i.c.v. infusion decreases the expression of POMC in rats and mice [65], [66]. Additionally, elevated NPY concentrations may also oppose the inhibitory effect of leptin in AGRP levels since NPY can up-regulate AGRP immunoreactivity, as shown in hypothalamic explants [67]. It should also be considered that viral expression of NPY is occurring in ARC neurons with distinct phenotypes, which could be further contributing to the deregulation of feeding circuits and ineffectiveness of peripheral compensatory mechanisms. For example, the expression of NPY in anorexigenic ARC neurons, such as POMC or neurotensin neurons, which are activated by circulating leptin [63], [68], may be inducing a further increase in NPY expression in these neurons.

Another factor contributing to the inconsistent levels of ARC neuropeptides observed in hyperleptinemic ARC-NPY rats may be the development of leptin resistance by hypothalamic neurons. In fact, the number of leptin-activated neurons in the ARC is significantly reduced in DIO mice, positioning the ARC as a major site of leptin resistance [69], [70]. Moreover, leptin responsiveness by NPY/AGRP and POMC neurons is drastically decreased in DIO mice [71] and after chronic leptin treatment [13], [72].

Interestingly, in these obese rats, the levels of CART were moderately increased compared to controls, suggesting that CART expression is responding to the high levels of leptin.

On the peripheral level, obese ARC-NPY rats revealed an increase in the number of large adipocytes along with a decrease in mature adipocytes marker PPAR-gamma-2 levels in white adipose tissue (WAT), suggesting a dysfunction in the adipocytes of severe obese rats. This dysfunction may be related to hypoxia that occurs in the adipose tissue of obese mice proportionally to body fat gain [73], [74]. Hypoxia promotes a decrease in PPAR-gamma-2 expression in adipocytes [75], [76], and can explain the down-regulation of this protein in our model. Moreover, obese ARC-NPY rats presented high NPY serum levels as compared to controls underlie the contribution of sympathetic NPY to the development of obesity [77], [78], [79].

### Moderate down-regulation of ARC NPY

The silencing strategy applied in this work, AAV vectors delivering synthetic microRNA that target NPY, could only achieve a 50% down-regulation in the ARC NPY immunoreactivity. These subtle silencing levels may be owing to a non total transduction of the NPY expressing cells by the AAV vectors, despite the fact that EGFP expressing cells were found throughout the length of the ARC (data not shown). Moreover, the non-transduced NPY neurons may be overriding to compensate for those transduced with the miR-NPY, thus decreasing the silencing levels. Nevertheless, the 50% down-regulation of ARC NPY expression meets our aim of obtaining a modest modulation of NPY. In fact, leptin and insulin can have an equivalent or stronger inhibitory effect in the ARC NPY expression, achieving about 0.5 to 1.5-fold decrease of ARC NPY levels in normal, fasted or diabetic rats [11], [15], [16].

In the present study, sustained inhibition of NPY expression in the ARC of adult rats was not sufficient to cause alterations on food intake and body weight gain in basal conditions. These results may seem surprisingly weak when compared to the severe phenotype observed upon ARC NPY overexpression, but ARC NPY down-regulation does not necessarily represents the opposite of NPY overexpression. In fact, overexpression of an anorexigenic neuropeptide, as for example POMC or its cleavage product of α-melanocyte stimulating hormone (α-MSH), would correspond to the opposite experiment of NPY overexpression. As reported by others, AAV-mediated overexpression of POMC in the ARC results in decreased food intake, body weight and adiposity in age-related obese rats and Zucker obese rats, with an improvement of the metabolic parameters [80], [81].

Deletion of NPY in embryos or neonatal mice [47], [48], progressive loss of hypothalamic NPY neurons [82] and, as shown in this study, moderate down-regulation of NPY expression in the ARC of adult rats does not induce alterations on basal feeding behavior. However, our work demonstrate that the response to challenging situations, like food deprivation, is impaired such that fasting-induced hyperphagia or fasting-induced decrease on locomotor activity are absent in rats with down-regulation of ARC NPY levels. Our findings obtained from specific down-regulation of NPY in ARC neurons confirms that fasting-induced responses are mediated by the increase of NPY expression in the ARC neurons [4], [21].

Nevertheless, our results are not in accordance with previous studies reporting reduced food intake and cumulative weight gain after down-regulation of ARC NPY by anti-sense oligonucleotides (AS-NPY) [83]. Despite the fact that both studies use the same vector (AAV), other technical aspects may explain the divergent observation. First, the silencing strategies used in the studies are different. In fact, Gardiner et al. [83] delivered anti-sense oligonucleotides under the control of an ubiquitous CMV promoter. Anti-sense oligonucleotides inhibit the transcription of mRNA to protein by physically blocking the access of ribosome to mRNA and/or activation of RNase H, which will cleave the DNA-RNA heteroduplex [84]. In opposition, we delivered artificial microRNAs under the control of a neuron-specific polymerase II promoter. The artificial microRNAs take part of the RNA-induced silencing complex (RISC) and bind to the target mRNA in a sequence-specific manner, promoting gene silencing. This novel RNA interference mechanism has proven to mitigate RNAi-mediated toxicity in the brain [85]. Therefore, the more specific (expression of microRNA exclusive in neurons) strategy employed in the present work may account for some of the reported differences. Finally, we evaluated the ARC NPY down-regulation by quantification of NPY immunoreactivity in brain sections while Gardiner et al. [83] measured the levels of NPY released from hypothalamic explants. Since the techniques used to assess the NPY down-regulation are different, we cannot compare accurately the silencing levels achieved with the two strategies.

Down-regulation of ARC NPY was not compensated with alterations on other ARC neuropeptides (immunoreactivity for AGRP, POMC and CART was unchanged compared to controls) which can be explained by two reasons: 1) compensatory changes that may occur to keep food intake quantities are not mediated by alterations in the expression of these neuropeptides, or 2) sustained 50% decrease of NPY levels is not sufficient to produce compensatory alterations on these neuropeptides.

### Conclusions

All together, our data shows that sustained moderate up-regulation of orexigenic NPY in the ARC results in diurnal over-feeding and disruption of the compensatory feeding responses. In addition, specific down-regulation of this orexigenic signal inhibits the fasting induced responses. These results highlight the importance of the physiological ARC NPY levels oscillations on feeding regulation and fasting response, as well as in body weight preservation and, possibly, in the prevention of obesity. The findings here reported are important for the design of therapeutic interventions for obesity that include the NPY.

## Methods

### Animals

Male Wistar rats were purchased from Charles River Laboratories. Rats were individually housed under a 12 h light/dark cycle in a temperature/humidity controlled room with *ad libitum* access to water and a standard chow diet. All experimental procedures were performed in accordance with the European Union Directive 86/609/EEC for the care and use of laboratory animals. Moreover, all the people working with animals have received appropriate education (FELASA course) as required by the Portuguese authorities. In addition, animals are housed in our licensed animal facility (international Animal Welfare Assurance number 520.000.000.2006). The present study is included in a project approved and financed by the Portuguese science foundation that approved the animal experimentation described. CNC animal experimentation board approved the utilization of animals for this project (reference PTDC/SAU-FCF/099082/2008).

### Construction of NPY and microRNA expression vectors

Rat *NPY* cDNA from p46F06444D-NPY (RZPD) was cloned into AAV back-bone. Three microRNA (miR) expression vectors were constructed using the BLOCK-iT Pol II miR RNAi Expression Vector Kit (Invitrogen). Oligonucleotides targeting the rat NPY mRNA were designed and cloned into the pcDNA6.2GW/EmGFP-miR vector. The miR control plasmids pcDNA6.2GW/EmGFP-miR-control (random sequence) was provided with the BLOCK-iT Pol II miR validated miRNA Control Vectors kit (Invitrogen). The sense sequences of the cloned microRNAs was: miR-1, 5′TGCTGCAAACACACGAGCAGGGATAGGTTTTGGCCACTGACTGACCTATCCCTTCGTGTGTTTG3′;miR-2, 5′TGCTGAGAATGCCCAAACACACGAGCGTTTTGGCCACTGACTGACGCTCGTGTTTGGGCATTCT3′; miR-3, 5′TGCTGTGAGATTGATGTAGTGTCGCAGTTTTGGCCACTGACTGACTGCGACACCATCAATCTCA3′; and miR-ctr, 5′TGCTGAAATGTACTGCGCGTGGAGACGTTTTGGCCACTGACTGACGTCTCCACGCAGTACATTT3′. The EGFP and miR sequences were cloned into the AAV back-bone by performing a BP/LR recombination.

### *In vitro* screen of microRNAs

For the validation of NPY vector and synthetic microRNA, HEK 293 cells (American Type Culture Collection, ATCC CRL-11268, Manassas, USA, ATTC) were co-transfected with pAAV-hSyn-NPY and one of the pAAV-hSyn-miR plasmids, in a 1∶1 ratio, using standard calcium phosphate method. Forty-eight hours after transfection, cell lysates were obtained and thirty micrograms of protein were used for 16% polyacrylamide gel Tricine-SDS electrophoresis. Protein samples were transferred to a 0.22 µm nitrocellulose membrane (Applichem). The membrane was incubated with anti-NPY antibody (1∶3000, Sigma) overnight at 4°C followed by incubation with alkaline phosphatase-linked antibody (1∶10000; GE Healthcare) and detection by ECF substrate. NPY and tubulin expression levels were calculated by densitometry analysis (Bio-Rad Fluor MaxS software).

### Injection of AAV into the rat arcuate nucleus

Recombinant AAV particles were generated as described before [86], [87]. The AAV plasmids were packaged into an AAV-1/2 chimerical capsid using AAV-1 and AAV-2 packaging plasmids in a 50∶50 ratio. The ability of AAV to transduce NPY positive cells was evaluated by co-localization of NPY immunostaining (anti-NPY 1∶500; Sigma) with EGFP expression in rat primary cortical neurons [88] and visualization on a confocal microscope (Zeiss).

Eighteen male Wistar rats weighing 210–230 g were randomly divided into three groups (n = 6 rats per group). Rats were anesthetized with an intraperitoneal injection of ketamine/xylazine (100 mg/kg and 20 mg/kg, respectively) and placed on a stereotaxic frame. Injection was performed bilaterally into the ARC following the coordinates: 2.60 mm posterior to the bregma, 0.5 mm lateral to the middle line and 9.55 mm ventral to the brain surface. The control and the NPY silencing groups received 15.0×10^9^ v.g./side of AAV-hSyn-EGFP-miR-ctr and AAV-hSyn-EGFP-miR-NPY, respectively (v.g., viral genomes), in a final volume of 2 µl/side. The NPY overexpression group received 9.0×10^9^ v.g./side of AAV-hSyn-NPY, in a final volume of 2.5 µl/side. Injection was performed at a rate of 0.5 µl/min with a 10 µl-Hamilton syringe attached to an automatic Pump Controller (WPI). Needle was kept in place for 10 minutes after end of infusion. Rats were allowed to recover for 6 days. The ARC was defined using The Paxino's Rat Brain Atlas and AAV infection was anatomically localized by EGFP-expressing neurons in the ARC.

### Food intake and body weight gain analysis

Rats were individually housed and monitored for eight-weeks after AAV injection. Body weight was measured twice a week and food intake once a week; during the food intake studies rats were housed in hanging wire mesh cages. The testes to evaluate the feeding response to food deprivation (FD) were carried as described: basal food intake was measured on the 24-hours before FD and then rats were food deprived for 36-hours. Next, rats were re-fed with standard chow and food intake was measured on the following 12-hours. Re-feeding was performed on different periods of the day, precisely in the beginning of light period or dark period, in the fourth and fifth week, respectively. Rats were allowed to recover for six days with *ad libitum* access to food before the next test. Activity was assessed on an activity box and number of crossings was recorded, on basal condition and after 24-hours on food deprivation, in the fifth week. Rats were always kept with *ad libitum* access to water.

### Collection of blood and tissues

Non-fasted rats were sacrificed 56 days after AAV injection by lethal dose of Sodium Thiopental (Braun) and intracardially perfused with 4% paraformaldehyde (PFA). Brains were removed and epididymal fat was isolated and weighted. Samples from epididymal adipose tissue were collected and immediately frozen in liquid nitrogen or fixed in PFA. Prior to perfusion, blood was collected and serum was separated by centrifugation (2 000 g, 15 minutes).

### Serum analysis

Glycemia was measured using Accu-Check Blood Glucose Sensor (Roche). ELISA kits were used to measure serum levels of leptin and insulin (both from Millipore) and NPY peptide (Phonenix Pharmaceuticals) following the manufacturer's instructions. Other serum parameters were measured on an automated Synchron Clinical System (Beckman Coulter).

### Adipose tissue analysis

Fixed epididymal adipose tissue samples were sectioned to twenty micrometers on a cryostat. Adipocytes auto-fluorescence was visualized. For each rat, adipocytes average diameter was determined on a 0.75 mm^2^ area, using the AxioVision software. The obtained diameter values were grouped by size range and adipocytes size distribution was determined as percentage of total number of adipocytes evaluated. Protein extracts were obtained from epididymal adipose tissue samples by homogenization of tissue samples in RIPA lyses buffer, followed by sonication. Afterwards, the homogenates were centrifuged and separated from the fat layer with 23G needle. Fifteen micrograms of protein were used for 12% polyacrylamide gel Glycine-SDS electrophoresis. After blotting, the membrane was incubated with rabbit anti-PPAR-gamma antibody (1∶500; Santa Cruz) and mouse anti-actin (1∶5000, Sigma) following the protocol described above. Protein expression levels were calculated by densitometry analysis (Bio-Rad Fluor MaxS software).

### Immunohistochemistry of brain slices

Immunohistochemistry was performed on brain sections using the following primary antibodies: rabbit anti-NPY (1∶6000, Sigma), rabbit anti-AGRP (1∶500, Phoenix Pharmaceuticals), rabbit anti-POMC (1∶500, Phoenix Pharmaceuticals) and rabbit anti-CART (1∶4000, Phoenix Pharmaceuticals). Briefly, thirty micrometers brain coronal sections were blocked in PBS containing 10% newborn goat serum (NGS; Gibco) and 0.3% Triton X-100 (Sigma) and incubated in primary antibody overnight at 4°C. Sections were then incubated with goat anti-rabbit Alexa-Flour 594 (1∶200, Invitrogen) for one hour at room temperature. Brain sections were observed on a Zeiss Axiovert microscope.

### Determination of hypothalamic neuropeptides immunoreactivity

Peptides immunoreactivity was determined on photographs of immunohistochemical sections as described above. The ARC was defined using The Paxino's Rat Brain Atlas. Sections of approximately equal spacing were sampled between Bregma −2.00 to −3.50 for NPY and AGRP immunoreactivity determination and Bregma −2.30 to −3.30 for POMC and CART immunoreactivity determination. Multiple including was avoided by analyzing non adjacent sections. For each peptide evaluated all sections were simultaneously subjected to immunohistochemistry procedure and to photographs acquisition. Images were taken on a Zeiss Axiovert microscope with automated stage. An area that includes the ARC or the nuclei DMH and VMH was encircled and the mean grey value was measured for both hemispheres separately using the ImageJ software. For each rat evaluated, mean grey values from a total of 6 hemispheres for NPY and AGRP and 4 hemispheres for POMC and CART were obtained. Immunoreactivity was calculated by dividing the mean grey value (arbitrary units) by the exposure time. Due to NPY expression modulation, two different exposure times had to be used when acquiring NPY immunostaining photographs. For the other peptides, photographs were taken with the same exposure time.

### Statistical methods

Results are expressed as mean and SEM. Neuropeptides immunoreactivity, western-blot densitometry, epididymal fat pad weight and serum parameters were analyzed by one-way ANOVA followed by post hoc Bonferroni test. Cumulative body weight gain, food intake and adipocytes size distribution were analyzed by two-way ANOVA. Food intake and locomotor activity under fasting conditions were analyzed by paired T-test.
